# Early Phase of the COVID-19 Outbreak in Hungary and Post-Lockdown Scenarios

**DOI:** 10.3390/v12070708

**Published:** 2020-06-30

**Authors:** Gergely Röst, Ferenc A. Bartha, Norbert Bogya, Péter Boldog, Attila Dénes, Tamás Ferenci, Krisztina J. Horváth, Attila Juhász, Csilla Nagy, Tamás Tekeli, Zsolt Vizi, Beatrix Oroszi

**Affiliations:** 1 Bolyai Institute, University of Szeged, 6720 Szeged, Hungary; rost@math.u-szeged.hu (G.R.); nbogya@math.u-szeged.hu (N.B.); boldogpeter@gmail.com (P.B.); denesa@math.u-szeged.hu (A.D.); horvath.j.krisztina@nnk.gov.hu (K.J.H.); juhasz.attila@nfo.bfkh.gov.hu (A.J.); nagy.csilla@nfo.bfkh.gov.hu (C.N.); tekeli@math.u-szeged.hu (T.T.); zsvizi@math.u-szeged.hu (Z.V.); oroszi.beatrix@nnk.gov.hu (B.O.); 2 Physiological Controls Research Center, Óbuda University, 1034 Budapest, Hungary; ferenci.tamas@nik.uni-obuda.hu; 3 Department of Public Health, Government Office of Capital City Budapest, 1034 Budapest, Hungary

**Keywords:** COVID-19, epidemic, Hungary, under-ascertainment, SARS-CoV-2, compartmental model, reproduction number, intervention, case fatality rate

## Abstract

COVID-19 epidemic has been suppressed in Hungary due to timely non-pharmaceutical interventions, prompting a considerable reduction in the number of contacts and transmission of the virus. This strategy was effective in preventing epidemic growth and reducing the incidence of COVID-19 to low levels. In this report, we present the first epidemiological and statistical analysis of the early phase of the COVID-19 outbreak in Hungary. Then, we establish an age-structured compartmental model to explore alternative post-lockdown scenarios. We incorporate various factors, such as age-specific measures, seasonal effects, and spatial heterogeneity to project the possible peak size and disease burden of a COVID-19 epidemic wave after the current measures are relaxed.

## 1. Introduction

A cluster of pneumonia cases of unknown origin was detected in Wuhan city, the capital of Hubei Province, China, with a population of 11 million in December 2019. On 31 December 2019 China alerted the World Health Organization (WHO) China Country Office [1]. On 7 January 2020 the causative pathogen of the pneumonia outbreak was identified as a novel coronavirus, and, on 11 February, the WHO officially named the novel coronavirus as SARS-CoV-2 and the disease it causes as COVID-19.

SARS-CoV-2 infection quickly spread from China, where it emerged in December 2019, to Europe, where the first cases were confirmed on 24 January 2020 in France (where, later in April, COVID-19 was retrospectively confirmed for a patient hospitalized in late December 2019) [2,3]. Around the same time, on 27 January, the first infection in Germany was confirmed in Bavaria that led to a local outbreak. By February 19, 16 subsequent cases have been confirmed and 241 high-risk contacts have been identified via agile contact-tracing [4]. The first epidemic in Europe started in the Lombardy region of Italy with the first detection on 20 February 2020 [5]. The WHO Director-General declared the COVID-19 outbreak a Public Health Emergency of International Concern under International Health Regulations (2005) on 30January 2020 [6] and then a pandemic on 11 March 2020 [7]. By that time, the number of daily new cases of COVID-19 was over 100 in several countries, including Italy, France, and Germany.

Containment measures started in mid-March in most of the EU-EEA countries, which included physical distancing measures such as school and workplace closures, cancellation of public events, restrictions of gatherings, and stay-at-home requirements as a response to the pandemic, aiming to reduce transmission of SARS-CoV-2. These measures reflect strong effort to suppress, or at least to slow down COVID-19. Some governments hesitated to mount a broad containment response quickly when the virus first emerged and there were also differences in the severity of restrictions they put in place at first. As a result, the peak daily incidence varied substantially between EU/EEA countries during the first COVID-19 pandemic wave [8].

The emergence of the new coronavirus in Hungary did not happen until early March. Phylogenetic analysis revealed multiple and parallel introduction of the virus into the country [9]. By that time, diagnosis and treatment, surveillance, epidemiological investigation, management of close contacts, and diagnostic testing were being developed and put in place. The strategy of Hungary was to suppress the epidemic to avoid the overload of the health care system and excess deaths, so they decided on fast and strong measures right after community transmission was established in Italy and a pandemic was declared by the WHO.

Here, we provide the first epidemiological and statistical study of the early phase of the COVID-19 outbreak in Hungary, focusing on the period 4 March–10 May. Our analysis provides insights to understand how the first wave transpired in the country, and shows that the timely non-pharmaceutical interventions were successful. Since the control measures are being progressively relaxed, to explore possible future scenarios, we employ an age structured compartmental model. The model allows us to investigate the impact of contact reductions, age-specific measures, seasonality, and spatial heterogeneity on the transmission dynamics, healthcare demand, mortality, and overall disease burden.

## 2. Materials and Methods

### 2.1. Epidemiological Report

During the last week of January 2020, the National Public Health Center (hereinafter NPHC) issued the surveillance protocol of COVID-19 cases that included the case definitions of COVID-19 cases. Until mid May, the protocol was updated four times as the relevant WHO and/or European Centre for Disease Prevention and Control (ECDC) guidance was revised. According to the Hungarian protocol, suspected cases were to be reported by physicians to the local health authority and NPHC, followed by compulsory testing, 14-day isolation and monitoring, case investigation, and contact tracing. A person with laboratory confirmation (detection of SARS-CoV-2 by polymerase chain reaction), irrespective of clinical signs and symptoms, was considered a confirmed COVID-19 case.

The primary data source regarding the COVID-19 outbreak in Hungary was the NPHC. Official announcements were published on a government hosted website [10] since 1 March, including epidemiological data, but not in a systematic way. The findings of our epidemiological analysis are detailed in Section 3.1.

### 2.2. Statistical Analysis

Statistical analysis of the available surveillance data—such as case counts and death counts—is an indispensable tool during outbreak response. It can reveal the current situation objectively, shed light on the effect of past interventions, uncover non-obvious aspects of the outbreak, and produce forecasts. It can also provide important input information to disease dynamics models.

This section summarizes the statistical analyses carried out, based on data from NPHC, during the early phase of the COVID-19 outbreak in Hungary, pointing out the most important tasks and methods.

All methods were implemented under the R statistical environment version 4.0.0 [11] using packages ggplot2 version 3.3.0 [12] for visualization, data.table version 1.12.8 [13] for data manipulation and shiny version 1.4.0.2 [14] for creating an interactive dashboard to carry out epidemiological analyses online (available in Hungarian [15]).

The full source code of this dashboard and related analysis is available at [16].

#### 2.2.1. Temporal Variation of the Effective Reproduction Number

Effective reproduction number ($R_{t}$), the average number of secondary cases per primary case for those primary cases who turn infectious on day *t*, was tracked in real time based on the daily number of reported new cases using the methods of Cori et al. [17] and that of Wallinga and Teunis [18], among the several methods aimed to estimate $R_{t}$ [19,20]. In brief, the method of Cori et al., is based on calculating the ratio of the actual number of infections on a day to the total infectiousness of all past cases on that day. Thus, it measures $R_{t}$ by assuming that infected individuals will infect in the future as if conditions remain unchanged. In contrast, the method of Wallinga and Teunis makes no such assumption; it uses a likelihood-based inference on the possible infection networks underlying the epidemic curve. The fundamental difference is that the method of Cori et al., solely uses past information (“backward looking approach”), due to which the result is sometimes called instantaneous reproduction number, while the Wallinga–Teunis method more closely corresponds to the concept of the usual definition of effective reproduction number; however, it requires future information in exchange (“forward looking approach”).

For a discussion on the relative merits of these two approaches, see [21,22]. The Wallinga–Teunis method was used with the addition of Cauchemez et al., who aimed to provide real-time estimation capability [23].

Both methods require—in addition to incidence data—information on the serial interval. Depending on the used dataset, different estimations of the serial interval have been published: a mean of 3.96 days was found in [24], and 6.6 days in [25]. Here, we assume an intermediate value following [26], where the mean and standard deviation (SD) of the serial interval were estimated at 4.7 days (95% CrI: 3.7, 6.0) and 2.9 days (95% CrI: 1.9, 4.9), respectively. (The serial interval is assumed to follow gamma distribution.) They also concluded that the serial interval of COVID-19 is close to or shorter than its median incubation period, which is coherent with our choice of parameters in the transmission dynamics model.

The estimation was carried out using R packages R0 version 1.2-6 [27,28] and EpiEstim version 2.2-2 [17,29].

#### 2.2.2. Adjusted Case Fatality Ratio

Case fatality rate (CFR) is defined as the (conditional) probability of death from a disease for those contracting the disease (for diseases where asymptomatic state also exists, infection fatality rate (IFR) is defined analogously) and is estimated as the ratio of cumulative deaths and cumulative cases. This definition, i.e.,

$$nCFR_{t}=\frac{\sum_{i=1}^{t}d_{i}}{\sum_{i=1}^{t}c_{i}}=\frac{D_{t}}{C_{t}},$$

where $c_{t}$ and $d_{t}$ are the daily, $C_{t}$ and $D_{t}$ are cumulative number of cases and deaths, respectively, on day *t* is however biased when used during the epidemic (thus the name crude/naive CFR or nCFR). The reason for this is that a proportion of cases counted in the denominator will die (in the future), thus they should have been counted in the numerator as well, but, as they’re not, the ratio underestimates the true value [30,31].

Fortunately, it is relatively easy to correct for this bias using information on the distribution of the diagnosis-to-death time [32,33]. The likelihood that the cumulative number of deaths on day *t* is $D_{t}$ is given by

∑i=1t∑j=0i−1ci−jfjDtπDt1−π∑i=1t∑j=0i−1ci−jfj−Dt,

where $f_{i}$ denotes the (conditional) probability that death happens on day *i* after onset (for those who die) and π stands for the true value of the CFR. This observation allows both maximum likelihood and Bayesian estimation for π using the observed series of $c_{i}$ and $d_{i}$, the latter of which was employed in the present study, using a Beta(1,1) (i.e., uniform) prior.

It was assumed that diagnosis-to-death time follows lognormal distribution with a mean of 13 days and a standard deviation of 12.7 days, as found by Linton et al. [34].

The Bayesian estimation was manually coded using the R package rstan version 2.19.3 [35]. A Markov chain Monte Carlo approach was used to carry out the estimation with No-U-Turn sampler, using 4 chains, 1000 warmup iterations and 2000 iterations for each chain.

#### 2.2.3. Estimation of the Ascertainment Rate

The CFR mentioned in the previous subsection is still not the true value of the fraction of fatal outcomes of all infections, as there is another source of bias, but this time leading to overestimation: the underascertainment of cases. This is a substantial issue now as a—precisely not yet known, but epidemiologically significant—fraction of the COVID-19 cases are asymptomatic or mildly symptomatic. Since in many countries testing was extended to contacts (and in a few instances, even random sampling was carried out), the confirmed cases include some asymptomatic cases as well.

However, the value of the estimated (corrected) IFR can also be used to estimate the ascertainment rate: by assuming that the IFR in reality takes a benchmark value (one derived from large-sample, well-designed studies accounting for underascertainment or sero-epidemiological surveys) and—crucially—assuming that the difference of the actual estimated IFR from that value is purely due to underascertainment. Then, the ascertainment rate can be obtained by simply dividing the assumed true value of the IFR with the actual estimated CFR [36]. Note that this might be a strong assumption, as it rules out that there is a real difference in the country’s IFR from the benchmark value; in particular, it rules out different virulence of the pathogen, different age- and comorbidity-composition in the country and different effect of the healthcare system on survival.

### 2.3. Transmission Model

Mathematical models have been developed to better understand the global spread [37] and the transmission dynamics of COVID-19 for many countries, including Australia [38], France [39], Germany [40], UK [41], and the USA [42,43]. Such models have been used to project the progress of the outbreak and to estimate the impact of control measures on reducing disease burden. The two most common approaches are compartmental models formulated as systems of ordinary differential equations, and agent based models used to generate an ensemble of stochastic simulations for possible outcomes.

Here, we establish a compartmental population model, adjusted to the specific characteristics of COVID-19, considering the following compartments. We denote by *S* the susceptibles, i.e., those who can be infected by the disease. Latents (*L*) are those who have already contracted the disease but do not show symptoms and are not infectious yet. In accordance with studies indicating that viral shedding peaks before the onset of symptoms [44], in our model, we have introduced the presymptomatic infected compartment $I_{p}^{}$ for those who do not have symptoms, but who already are capable of transmitting the disease to susceptibles. We divided the latent period into two compartments $L_{1}^{}$ and $L_{2}^{}$, thus, together with $I_{p}^{}$, the incubation period follows a hypoexponential distribution, having a shape matching empirical observations [45,46]. Since a large fraction of infected shows only mild or no symptoms, after the incubation period, we differentiate these individuals from those with symptoms. We assume a gamma-distributed infectious period with Erlang parameter m=3, similar to the SARS study [47], hence, we have three classes for both asymptomatic and symptomatic infectious individuals ($I_{a,1}^{},I_{a,2}^{},I_{a,3}^{}$ and $I_{s,1}^{},I_{s,2}^{},I_{s,3}^{}$, respectively). Individuals from the $I_{a,3}^{}$ compartment will all recover and hence proceed to the recovered class $R_{}^{}$. Immunity is assumed for those who have recovered from the disease, at least for the time scale of this modeling. Individuals from $I_{s,3}^{}$ may either recover without requiring hospital treatment (and thus move to $R_{}^{}$) or become hospitalized. It is of crucial importance to project the number of hospital beds and intensive care unit (ICU) beds needed; thus, in the model, we further differentiate symptomatically infected individuals who need hospital care and critical care, denoted by $I_{h}^{}$ and $I_{c}^{}$, respectively. We operate with the assumption that the healthcare system will not be overwhelmed, and thus disease-induced death is only considered from critical care that fits with the data obtained from NPHC. Hence, individuals from $I_{h}^{}$ will proceed to $R_{}^{}$ after recovery. Those from $I_{c}^{}$ with fatal outcome transit to the $D_{}^{}$ compartment. Those who are out of ICU and on the path to recovery are collected into the $I_{\mathrm{cr}}^{}$, from where they eventually recover and move to the $R_{}^{}$ class.

To take into account the different characteristics of the disease in various age groups, we stratified the Hungarian population into seven groups, corresponding to the available choices in the Hungarian online questionnaire for the assessment of changes in the number of contacts following the lockdown [48]. The compartments listed above corresponding to the different age groups are denoted by an upper index i∈1,…,7. Accordingly, all of our parameters can be calibrated age-specifically.

**Figure 1:**
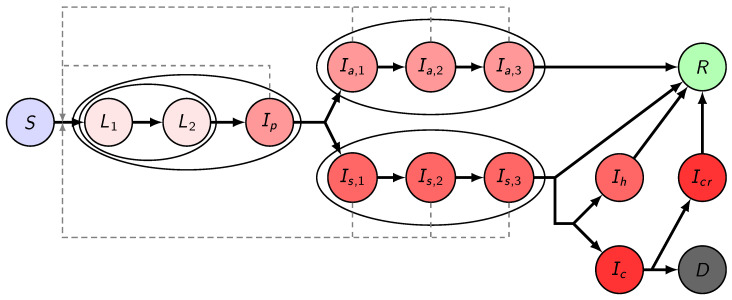
Transmission diagram. Different shades of red denote infected compartments. A darker shade corresponds to a more severe state of the disease: those in compartments with the lightest shade do not transmit the disease, those in compartments with the darkest shade are in critical status. Black solid arrows denote the possible ways of transition from one compartment to another. Gray dashed arrows show possible ways of infection. Compartments being grouped together in ellipses stand for different stages of the same status.

The transmission rates from age group *k* to age group *i* are denoted by $\beta_{j}^{\left(k,i\right)}$, with j∈{p,a,s}, where the three subscripts p,a,s stand for presymptomatic, asymptomatic, and symptomatic infected, respectively. The parameters described in the following all have an upper index *i* which stands for the corresponding age group. A fraction $p_{}^{i}$ of exposed people will not show symptoms during his/her infection, while $\left(1-p_{}^{i}\right)$ will develop symptoms. The average length of the incubation period is ${\left(\alpha_{L,1}^{i}\right)}^{-1}+{\left(\alpha_{L,2}^{i}\right)}^{-1}+{\left(\alpha_{p}^{i}\right)}^{-1}$ days, with the transition rates $\alpha_{L,1}^{i},\alpha_{L,2}^{i},\alpha_{p}^{i}$, respectively. Similarly, the average infectious period of asymptomatic and symptomatic infected individuals are ${\left(\gamma_{a,1}^{i}\right)}^{-1}+{\left(\gamma_{a,2}^{i}\right)}^{-1}+{\left(\gamma_{a,3}^{i}\right)}^{-1},$ and ${\left(\gamma_{s,1}^{i}\right)}^{-1}+{\left(\gamma_{s,2}^{i}\right)}^{-1}+{\left(\gamma_{s,3}^{i}\right)}^{-1}$, with the corresponding transition rates, respectively. A fraction $h^{i}$ of the infectious compartment $I_{s,3}^{i}$ will be hospitalized, the remaining fraction $1-h^{i}$ will recover without hospital care. Out of those who need hospitalization, a fraction $\xi^{i}$ needs intensive care. For the hospitalized classes $I_{h}^{i}$, $I_{c}^{i}$, $I_{\mathrm{cr}}^{i}$, the average time spent in these compartments is given as ${\left(\gamma_{h}^{i}\right)}^{-1}$, ${\left(\gamma_{c}^{i}\right)}^{-1}$ and ${\left(\gamma_{\mathrm{cr}}^{i}\right)}^{-1}$, respectively. A fraction $\mu^{i}$ of those leaving the $I_{c}^{i}$ compartment will die due to the disease, while the remaining fraction will proceed to the $I_{\mathrm{cr}}^{i}$ class. The transmission dynamics of our model for one age group is illustrated in Figure 1.

The governing system of differential Equation (1) of our model can be found in Section 2.3.1. The model parameters with references are detailed in Section 2.3.2. A further important component of our model is the contact matrix, describing social mixing between the age groups, which can be found in Section 2.3.3. The elements of the contact matrix are included via the different transmission terms $\beta_{j}^{\left(k,i\right)}$. Reproduction numbers are calculated using the next generation matrix method in Section 2.3.4. We discuss the application and, then, some limitations of this model in Section 2.3.5 and Section 2.3.6, respectively. The codes were implemented in Wolfram Mathematica and are available at [49].

#### 2.3.1. The Governing Equations of the Transmission Model

The governing equations of the transmission model described in Section 2.3 take the form

(1) $$\begin{array}{cc} {S_{}^{i}}^{\prime }\left(t\right)= & -\frac{S_{}^{i}\left(t\right)}{N_{}^{i}\left(t\right)}\sum_{k\in \{1,\ldots ,7\}}\left[\beta_{p}^{\left(k,i\right)}I_{p}^{k}\left(t\right)+\sum_{j\in \{a,s\}\times \{1,2,3\}}\beta_{j}^{\left(k,i\right)}I_{j}^{k}\left(t\right)\right],\\ {L_{1}^{i}}^{\prime }\left(t\right)= & \frac{S_{}^{i}\left(t\right)}{N_{}^{i}\left(t\right)}\sum_{k\in \{1,\ldots ,7\}}\left[\beta_{p}^{\left(k,i\right)}I_{p}^{k}\left(t\right)+\sum_{j\in \{a,s\}\times \{1,2,3\}}\beta_{j}^{\left(k,i\right)}I_{j}^{k}\left(t\right)\right]-\alpha_{L,1}^{i}L_{1}^{i}\left(t\right),\\ {L_{2}^{i}}^{\prime }\left(t\right)= & \alpha_{L,1}^{i}L_{1}^{i}\left(t\right)-\alpha_{L,2}^{i}L_{2}^{i}\left(t\right),\\ {I_{p}^{i}}^{\prime }\left(t\right)= & \alpha_{L,2}^{i}L_{2}^{i}\left(t\right)-\alpha_{p}^{i}I_{p}^{i}\left(t\right),\\ {I_{a,1}^{i}}^{\prime }\left(t\right)= & p_{}^{i}\alpha_{p}^{i}I_{p}^{i}\left(t\right)-\gamma_{a,1}^{i}I_{a,1}^{i}\left(t\right),\\ {I_{a,2}^{i}}^{\prime }\left(t\right)= & \gamma_{a,1}^{i}I_{a,1}^{i}\left(t\right)-\gamma_{a,2}^{i}I_{a,2}^{i}\left(t\right),\\ {I_{a,3}^{i}}^{\prime }\left(t\right)= & \gamma_{a,2}^{i}I_{a,2}^{i}\left(t\right)-\gamma_{a,3}^{i}I_{a,3}^{i}\left(t\right),\\ {I_{s,1}^{i}}^{\prime }\left(t\right)= & \left(1-p_{}^{i}\right)\alpha_{p}^{i}I_{p}^{i}\left(t\right)-\gamma_{s,1}^{i}I_{s,1}^{i}\left(t\right),\\ {I_{s,2}^{i}}^{\prime }\left(t\right)= & \gamma_{s,1}^{i}I_{s,1}^{i}\left(t\right)-\gamma_{s,2}^{i}I_{s,2}^{i}\left(t\right),\\ I_{s,3}^{i}\left(t\right)= & \gamma_{s,2}^{i}I_{s,2}^{i}\left(t\right)-\gamma_{s,3}^{i}I_{s,3}^{i}\left(t\right),\\ {I_{h}^{i}}^{\prime }\left(t\right)= & h_{}^{i}\left(1-\xi_{}^{i}\right)\gamma_{s,3}^{i}I_{s,3}^{i}\left(t\right)-\gamma_{h}^{i}I_{h}^{i}\left(t\right),\\ {I_{c}^{i}}^{\prime }\left(t\right)= & h_{}^{i}\xi_{}^{i}\gamma_{s,3}^{i}I_{s,3}^{i}\left(t\right)-\gamma_{c}^{i}I_{c}^{i}\left(t\right),\\ {I_{\mathrm{cr}}^{i}}^{\prime }\left(t\right)= & \left(1-\mu_{}^{i}\right)\gamma_{c}^{i}I_{c}^{i}\left(t\right)-\gamma_{\mathrm{cr}}^{i}I_{\mathrm{cr}}^{i}\left(t\right),\\ {R_{}^{i}}^{\prime }\left(t\right)= & \gamma_{a,3}^{i}I_{a,3}^{i}\left(t\right)+\left(1-h_{}^{i}\right)\gamma_{s,3}^{i}I_{s,3}^{i}\left(t\right)+\gamma_{h}^{i}I_{h}^{i}\left(t\right)+\gamma_{\mathrm{cr}}^{i}I_{\mathrm{cr}}^{i}\left(t\right),\\ {D_{}^{i}}^{\prime }\left(t\right)= & \mu_{}^{i}\gamma_{c}^{i}I_{c}^{i}\left(t\right), \end{array}$$

where the index i∈{1,…,7} represents the corresponding age group.

Next, we add the spatial locations of the population to the previous model. The population is divided into patches, where each patch represents a separate geographic region. Within each region, we use the same compartmental model (but possibly with different parameters), and we also include spatial movement of individuals between the patches. The governing equations of such a metapopulation model, where $p_{1}\in \left\{1,2,\ldots ,\#\mathrm{patches}\right\}$ are

(2) $$\begin{array}{cc} {S_{p_{1}}^{i}}^{\prime }\left(t\right)= & -\frac{S_{p_{1}}^{i}\left(t\right)}{N_{p_{1}}^{i}\left(t\right)}\sum_{k\in \{1,\ldots ,7\}}\left[\beta_{p_{1},p}^{\left(k,i\right)}I_{p_{1},p}^{k}\left(t\right)+\sum_{j\in \{a,s\}\times \{1,2,3\}}\beta_{p_{1},j}^{\left(k,i\right)}I_{p_{1},j}^{k}\left(t\right)\right]\\ \; & \;+\sum_{p_{2}=1,p_{2}\neq p_{1}}^{\#\mathrm{patches}}\left(t_{p_{2},p_{1}}^{i}S_{p_{2}}^{i}-t_{p_{1},p_{2}}^{i}S_{p_{1}}^{i}\right),\\ {L_{p_{1},1}^{i}}^{\prime }\left(t\right)= & \frac{S_{p_{1}}^{i}\left(t\right)}{N_{p_{1}}^{i}\left(t\right)}\sum_{k\in \{1,\ldots ,7\}}\left[\beta_{p_{1},p}^{\left(k,i\right)}I_{p_{1},p}^{k}\left(t\right)+\sum_{j\in \{a,s\}\times \{1,2,3\}}\beta_{p_{1},j}^{\left(k,i\right)}I_{p_{1},j}^{k}\left(t\right)\right]-\alpha_{p_{1},L,1}^{i}L_{p_{1},1}^{i}\left(t\right)\\ \; & \;+\sum_{p_{2}=1,p_{2}\neq p_{1}}^{\#\mathrm{patches}}\left(t_{p_{2},p_{1}}^{i}L_{p_{2},1}^{i}-t_{p_{1},p_{2}}^{i}L_{p_{1},1}^{i}\right),\\ {L_{p_{1},2}^{i}}^{\prime }\left(t\right)= & \alpha_{p_{1},L,1}^{i}L_{p_{1},1}^{i}\left(t\right)-\alpha_{p_{1},L,2}^{i}L_{p_{1},2}^{i}\left(t\right)+\sum_{p_{2}=1,p_{2}\neq p_{1}}^{\#\mathrm{patches}}\left(t_{p_{2},p_{1}}^{i}L_{p_{2},2}^{i}-t_{p_{1},p_{2}}^{i}L_{p_{1},2}^{i}\right),\\ {I_{p_{1},p}^{i}}^{\prime }\left(t\right)= & \alpha_{p_{1},L,2}^{i}L_{p_{1},2}^{i}\left(t\right)-\alpha_{p_{1},p}^{i}I_{p_{1},p}^{i}\left(t\right)+\sum_{p_{2}=1,p_{2}\neq p_{1}}^{\#\mathrm{patches}}\left(t_{p_{2},p_{1}}^{i}I_{p_{2},p}^{i}-t_{p_{1},p_{2}}^{i}I_{p_{1},p}^{i}\right),\\ {I_{p_{1},a,1}^{i}}^{\prime }\left(t\right)= & p_{p_{1}}^{i}\alpha_{p_{1},p}^{i}I_{p_{1},p}^{i}\left(t\right)-\gamma_{p_{1},a,1}^{i}I_{p_{1},a,1}^{i}\left(t\right)+\sum_{p_{2}=1,p_{2}\neq p_{1}}^{\#\mathrm{patches}}\left(t_{p_{2},p_{1}}^{i}I_{p_{2},a,1}^{i}-t_{p_{1},p_{2}}^{i}I_{p_{1},a,1}^{i}\right),\\ {I_{p_{1},a,2}^{i}}^{\prime }\left(t\right)= & \gamma_{p_{1},a,1}^{i}I_{p_{1},a,1}^{i}\left(t\right)-\gamma_{p_{1},a,2}^{i}I_{p_{1},a,2}^{i}\left(t\right)+\sum_{p_{2}=1,p_{2}\neq p_{1}}^{\#\mathrm{patches}}\left(t_{p_{2},p_{1}}^{i}I_{p_{2},a,2}^{i}-t_{p_{1},p_{2}}^{i}I_{p_{1},a,2}^{i}\right),\\ {I_{p_{1},a,3}^{i}}^{\prime }\left(t\right)= & \gamma_{p_{1},a,2}^{i}I_{p_{1},a,2}^{i}\left(t\right)-\gamma_{p_{1},a,3}^{i}I_{p_{1},a,3}^{i}\left(t\right)+\sum_{p_{2}=1,p_{2}\neq p_{1}}^{\#\mathrm{patches}}\left(t_{p_{2},p_{1}}^{i}I_{p_{2},a,3}^{i}-t_{p_{1},p_{2}}^{i}I_{p_{1},a,3}^{i}\right),\\ {I_{p_{1},s,1}^{i}}^{\prime }\left(t\right)= & \left(1-p_{p_{1}}^{i}\right)\alpha_{p_{1},p}^{i}I_{p_{1},p}^{i}\left(t\right)-\gamma_{p_{1},s,1}^{i}I_{p_{1},s,1}^{i}\left(t\right)+\sum_{p_{2}=1,p_{2}\neq p_{1}}^{\#\mathrm{patches}}\left(t_{p_{2},p_{1}}^{i}I_{p_{2},s,1}^{i}-t_{p_{1},p_{2}}^{i}I_{p_{1},s,1}^{i}\right),\\ {I_{p_{1},s,2}^{i}}^{\prime }\left(t\right)= & \gamma_{p_{1},s,1}^{i}I_{p_{1},s,1}^{i}\left(t\right)-\gamma_{p_{1},s,2}^{i}I_{p_{1},s,2}^{i}\left(t\right)+\sum_{p_{2}=1,p_{2}\neq p_{1}}^{\#\mathrm{patches}}\left(t_{p_{2},p_{1}}^{i}I_{p_{2},s,2}^{i}-t_{p_{1},p_{2}}^{i}I_{p_{1},s,2}^{i}\right),\\ I_{p_{1},s,3}^{i}\left(t\right)= & \gamma_{p_{1},s,2}^{i}I_{p_{1},s,2}^{i}\left(t\right)-\gamma_{p_{1},s,3}^{i}I_{p_{1},s,3}^{i}\left(t\right)+\sum_{p_{2}=1,p_{2}\neq p_{1}}^{\#\mathrm{patches}}\left(t_{p_{2},p_{1}}^{i}I_{p_{2},s,3}^{i}-t_{p_{1},p_{2}}^{i}I_{p_{1},s,3}^{i}\right),\\ {I_{p_{1},h}^{i}}^{\prime }\left(t\right)= & h_{p_{1}}^{i}\left(1-\xi_{p_{1}}^{i}\right)\gamma_{p_{1},s,3}^{i}I_{p_{1},s,3}^{i}\left(t\right)-\gamma_{p_{1},h}^{i}I_{p_{1},h}^{i}\left(t\right),\\ {I_{p_{1},c}^{i}}^{\prime }\left(t\right)= & h_{p_{1}}^{i}\xi_{p_{1}}^{i}\gamma_{p_{1},s,3}^{i}I_{p_{1},s,3}^{i}\left(t\right)-\gamma_{p_{1},c}^{i}I_{p_{1},c}^{i}\left(t\right),\\ {I_{p_{1},\mathrm{cr}}^{i}}^{\prime }\left(t\right)= & \left(1-\mu_{p_{1}}^{i}\right)\gamma_{p_{1},c}^{i}I_{p_{1},c}^{i}\left(t\right)-\gamma_{p_{1},\mathrm{cr}}^{i}I_{p_{1},\mathrm{cr}}^{i}\left(t\right),\\ {R_{p_{1}}^{i}}^{\prime }\left(t\right)= & \gamma_{p_{1},a,3}^{i}I_{p_{1},a,3}^{i}\left(t\right)+\left(1-h_{p_{1}}^{i}\right)\gamma_{p_{1},s,3}^{i}I_{p_{1},s,3}^{i}\left(t\right)+\gamma_{p_{1},h}^{i}I_{p_{1},h}^{i}\left(t\right)+\gamma_{p_{1},\mathrm{cr}}^{i}I_{p_{1},\mathrm{cr}}^{i}\left(t\right)\\ \; & \;+\sum_{p_{2}=1,p_{2}\neq p_{1}}^{\#\mathrm{patches}}\left(t_{p_{2},p_{1}}^{i}R_{p_{2}}^{i}-t_{p_{1},p_{2}}^{i}R_{p_{1}}^{i}\right),\\ {D_{p_{1}}^{i}}^{\prime }\left(t\right)= & \mu_{p_{1}}^{i}\gamma_{p_{1},c}^{i}I_{p_{1},c}^{i}\left(t\right). \end{array}$$

#### 2.3.2. Model Parameters

We have chosen our model parameters based on comprehensive literature review and present them here, except the transmission rates $\beta_{s,\_}^{\left(k,i\right)}$ which are left for Section 2.3.4. For the incubation period, we assume hypoexponential (generalized Erlang) distribution with parameters (1.6, 1.6, 2). This way, the average incubation period is 5.2 days: the same length and very similar shape of the probability distribution function was estimated in [45], and this distribution has the observed concavity properties as well (see [46]). In addition, this estimation is consistent with [34], and such values have been used in [38,39,41,42]. The first 3.2 days are the latent period [38] and the past two days are the presymptomatic period [38], when transmission is already possible with similar rate as at symptom onset [44]. Therefore, we use the same transmission rates for the presymptomatic and symptomatic infectious periods. For the transmission rate of asymptomatic infected individuals, we use a reduction factor 0.5 [39,42,43].

For the length of infectious periods (both symptomatic and asymptomatic), we assume a gamma distribution with Erlang parameter 3 (coherent with the SARS study [47]), and an average length 3 days of infectivity. Although full recovery and viral shedding may take much longer, the infectiousness throughout the course of infection is mostly concentrated to this period [44,50]. The choice of 3 days is also justified by [44,51], who estimated that around 40% of transmissions occur during the presymptomatic period, and it is also within the range of infectious periods used by [39,42].

The average stay in hospital is assumed to be 10 days, in accordance with the seven days median reported in [52] using over 16,000 patients’ data in the UK. Similarly, the average duration of critical care is assumed to be 10 days, in accordance with the Intensive Care National Audit & Research Center (ICNARC) report [53]. Very similar numbers were reported in the US [54], and were used in other modeling studies [38,41,42]. For those who recover from intensive care, we assumed a 14-day hospitalized rehabilitation period.

The periods above associated with the average time an individual spends in each compartment over the course of the infection are age-independent and summarized in Table 1.

Next, we discuss the age-specific parameters, which are mostly related to the outcome of infections. We stratified the population into the following seven age groups: 0–4, 5–14, 15–29, 30–59, 60–69, 70–79, 80+ years old. Using the data from the Hungarian Central Statistical Office (KSH), we obtain the division shown in Table 2.

According to [55], a fraction 0.8 of infected children (under 18 years old) are asymptomatic or mild cases. This value was used in [42] as well. We set the probabilities of the infection following mild or asymptomatic course in an individual according to Weitz et al. [43].

The probabilities of hospitalization given infection $h_{}^{i}$ and of requiring intensive care in addition $\xi_{}^{i}$ are based on the work of Moss et al. [38]. The ratios of fatal outcomes $\mu_{}^{i}$ are derived from the ICNARC report [53] comprising 6720 ICU case reports from UK. All these age-dependent parameters are listed in Table 3.

#### 2.3.3. Contact Matrix

For creating our contact matrix $M_{\mathrm{cont}}$, we have utilized the work by Prem, Cook, and Jit [56], where the estimated matrices are written for 16 age groups, namely 0–4, 5–9, …, 70–74, 75+. As we have divided the Hungarian population into seven age groups, see Table 2, we aggregated the higher resolution data. First, we derived a symmetric matrix $M^{\mathrm{total}}$ with elements

$$m_{i,j}^{\mathrm{total}}=\frac{m_{i,j}p_{i}+m_{j,i}p_{j}}{2},$$

where $M=[m_{i,j}]$ is the original contact matrix and $\left[p_{i}\right]$ is the age distribution of Hungary for the same age groups as in [56]. Thus, $M^{\mathrm{total}}$ contains the total number of contacts among age groups in its upper triangular part (with values relative to the contact pattern in *M*). The total number of contacts, w.r.t. the age distribution used in our work, is then obtained by summing up the corresponding elements of this matrix of size 16×16 resulting in $M_{\mathrm{cont}}^{\mathrm{total}}$ of size 7×7. Finally, dividing element-wise each column of $M_{\mathrm{cont}}^{\mathrm{total}}$ by the aforementioned population vector given in 2 yields the following 7×7 contact matrix:

$$M_{\mathrm{cont}}=\left[\begin{array}{ccccccc} 2.85266 & 1.01297 & 0.979001 & 3.66453 & 0.759418 & 0.250632 & 0.153184\\ 0.498022 & 5.54587 & 1.36396 & 4.67031 & 0.715084 & 0.485708 & 0.255501\\ 0.273365 & 0.774654 & 6.17901 & 6.44003 & 0.594565 & 0.283886 & 0.13613\\ 0.420065 & 1.08891 & 2.64379 & 5.45488 & 0.905271 & 0.411717 & 0.185912\\ 0.271197 & 0.519408 & 0.760402 & 2.82023 & 1.66402 & 0.585505 & 0.162196\\ 0.139887 & 0.551395 & 0.567445 & 2.00466 & 0.915096 & 0.736195 & 0.144611\\ 0.165767 & 0.562374 & 0.527571 & 1.75507 & 0.491497 & 0.28038 & 0.55053 \end{array}\right].$$

**Figure 2:**
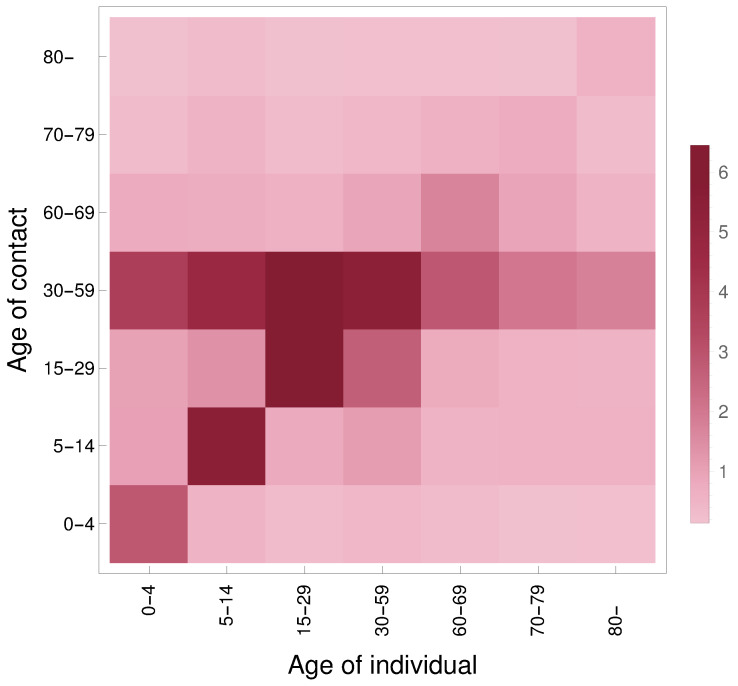
Heatmap of the contact matrix $M_{\mathrm{cont}}$. The image is rotated w.r.t. how $M_{cont}$ is indexed: the (1,1) element is in the bottom-left corner.

For more insight, we include its heatmap in Figure 2. Additional technical details are to be found in our source code available at [49].

#### 2.3.4. Transmission Rates and the Next Generation Matrix

Recall that we have assumed presymptomatic patients, which are members of classes $I_{p}^{i}$, to be as infectious as symptomatic patients. In addition, patients with no or mild symptoms (those in $I_{a}^{i}$) possess a transmission coefficient half of the baseline.

Thus, our task is to give reasonable estimates for the rates $\beta_{s,\_}^{\left(k,i\right)}$ corresponding to the transmission rate of the symptomatic individuals from age group *k* to group *i*. To that end, we follow the terminology and techniques of [57] to compute the Next Generation Matrix (NGM) and the baseline transmission rate $\beta_{0}$. Finally, the desired coefficients are obtained by taking into account the relative contact rates between age groups via the contact matrix presented in Section 2.3.3. We note that the probabilities $p^{i}$ have a special role during NGM computations as their effect is what ultimately specializes the resulting transmission rate matrix for COVID-19.

First, let us consider the infectious subsystem of (1), namely, equations describing ${L_{1}^{i}}^{\prime }\left(t\right)$, ${L_{2}^{i}}^{\prime }\left(t\right)$, ${I_{p}^{i}}^{\prime }\left(t\right)$, and ${I_{j}^{i}}^{\prime }\left(t\right)$ with j∈{a,s}×{1,2,3}, i∈{1,…,7}. Linearizing this w.r.t. the disease free equilibrium yields the linearized infectious subsystem:

$$X^{\prime }\left(t\right)=\left(T+\Sigma \right)\cdot X\left(t\right),$$

where the matrices T and Σ are referred to as the *transmission part* and *transitional part*, respectively; the state is described by

$$X\left(t\right)=\mathrm{transpose}\left(\left[\begin{array}{c} {\left\{L_{1}^{i}\left(t\right)\;\;L_{2}^{i}\left(t\right)\;\;I_{p}^{i}\left(t\right)\;\;{\left\{I_{a,n}^{i}\left(t\right)\right\}}_{n=1\ldots 3}\;\;{\left\{I_{s,n}^{i}\left(t\right)\right\}}_{n=1\ldots 3}\right\}}_{i=1\ldots 7} \end{array}\right]\right).$$

Recall that the transmission matrix T has the form

$$T=\left[\begin{array}{ccccccc} & & & T_{1} & & & \\ 0 & & \ldots & \left(2^{\mathrm{nd}}\;\mathrm{row}\right) & \ldots & & 0\\ & \vdots & & \vdots & & & \vdots \\ 0 & & \ldots & \left(8^{\mathrm{th}}\;\mathrm{row}\right) & \ldots & & 0\\ & & & T_{2} & & & \\ & \vdots & & \vdots & & & \vdots \end{array}\right],$$

where $T_{i}=\left[T_{1,i}\;\;T_{2,i}\;\;T_{3,i}\;\;T_{4,i}\;\;T_{5,i}\;\;T_{6,i}\;\;T_{7,i}\right]$ with

$$T_{k,i}=\left[\begin{array}{ccccccccc} 0 & 0 & \beta_{p}^{\left(k,i\right)} & \beta_{a,1}^{\left(k,i\right)} & \beta_{a,2}^{\left(k,i\right)} & \beta_{a,3}^{\left(k,i\right)} & \beta_{s,1}^{\left(k,i\right)} & \beta_{s,2}^{\left(k,i\right)} & \beta_{s,3}^{\left(k,i\right)} \end{array}\right].$$

On the other hand, the transitional matrix Σ is block diagonal with blocks

$$\Sigma_{i}=\left[\begin{array}{ccccccccc} -\alpha_{L,1}^{i} & 0 & 0 & 0 & 0 & 0 & 0 & 0 & 0\\ \alpha_{L,1}^{i} & -\alpha_{L,2}^{i} & 0 & 0 & 0 & 0 & 0 & 0 & 0\\ 0 & \alpha_{L,2}^{i} & -\alpha_{p}^{i} & 0 & 0 & 0 & 0 & 0 & 0\\ 0 & 0 & p_{}^{i}\alpha_{p}^{i} & -\gamma_{a,1}^{i} & 0 & 0 & 0 & 0 & 0\\ 0 & 0 & 0 & \gamma_{a,1}^{i} & -\gamma_{a,2}^{i} & 0 & 0 & 0 & 0\\ 0 & 0 & 0 & 0 & \gamma_{a,2}^{i} & -\gamma_{a,3}^{i} & 0 & 0 & 0\\ 0 & 0 & \left(1-p_{}^{i}\right)\alpha_{p}^{i} & 0 & 0 & 0 & -\gamma_{s,1}^{i} & 0 & 0\\ 0 & 0 & 0 & 0 & 0 & 0 & \gamma_{s,1}^{i} & -\gamma_{s,2}^{i} & 0\\ 0 & 0 & 0 & 0 & 0 & 0 & 0 & \gamma_{s,2}^{i} & -\gamma_{s,3}^{i} \end{array}\right]$$

for i=1,…,7.

Then, the NGM with large domain is given by

$$K_{L}=-T\Sigma^{-1}$$

and the NGM

$$K=E\;K_{L}\;\mathrm{transpose}\left(E\right)$$

follows with the, again, block diagonal E with $E_{i}=\left[\;1\;\;0\;\;0\;\;0\;\;0\;\;0\;\;0\;\;0\;\;0\;\right]$. The baseline transmission rate $\beta_{0}$ may be factored out from K as $\beta_{p}^{\left(k,i\right)}=\beta_{s}^{\left(k,i\right)}=\beta_{0}\cdot {\left(M_{\mathrm{cont}}\right)}_{k,i}$ and $\beta_{a}^{\left(k,i\right)}=\frac{1}{2}\beta_{s}^{\left(k,i\right)}=\beta_{0}\cdot \frac{{\left(M_{\mathrm{cont}}\right)}_{k,i}}{2}$. Hence, $K=\beta_{0}\cdot \hat{K}$, where $\hat{K}$ may be readily constructed and we can compute its spectral radius $\rho (\hat{K})$. Then, we obtain the baseline transmission rate using the assumed basic reproduction number $R_{0}$ as

$$\beta_{0}\cdot \rho \left(\hat{K}\right)=R_{0},$$

which is $\beta_{0}\approx 0.0462$ for $R_{0}=2.2$. Finally, the transmission rates $\beta_{s}^{\left(k,i\right)}$ are computed via the contact matrix $M_{\mathrm{cont}}$ as ${\left[\beta_{s}^{\left(k,i\right)}\right]}_{k,i}=\beta_{0}\;M_{\mathrm{cont}}=$

$$\left[\begin{array}{ccccccc} 0.131931 & 0.0468484 & 0.0452773 & 0.169479 & 0.035122 & 0.0115914 & 0.00708453\\ 0.0230328 & 0.256488 & 0.0630811 & 0.215995 & 0.0330716 & 0.0224633 & 0.0118165\\ 0.0126427 & 0.0358266 & 0.28577 & 0.297842 & 0.0274977 & 0.0131293 & 0.00629581\\ 0.0194274 & 0.0503605 & 0.122271 & 0.25228 & 0.0418674 & 0.0190413 & 0.00859815\\ 0.0125425 & 0.0240218 & 0.0351675 & 0.130431 & 0.0769585 & 0.0270787 & 0.00750132\\ 0.00646957 & 0.0255012 & 0.0262435 & 0.0927126 & 0.0423218 & 0.0340479 & 0.00668804\\ 0.00766648 & 0.026009 & 0.0243994 & 0.0811694 & 0.022731 & 0.0129672 & 0.0254612 \end{array}\right],$$

again, for $R_{0}=2.2$. For other scenarios, the final steps are altered to align with the desired reproduction number R, resulting in an appropriate β and then the scaled transmission rates $\beta_{s}^{\left(k,i\right)}$. We omit presenting all transmission matrices but give the computed baseline transmission rates in Table 4.

#### 2.3.5. Scenarios

We use the compartmental model described above to explore possible future scenarios, assuming widespread transmission in the population. In particular, we investigate the disease dynamics when different levels of general reductions of transmission, compared to the baseline, are in place. By manipulating the contact matrix, we investigate the effect of age-specific interventions, such as school closures and special measures aimed to protect the elderly.

Seasonality of respiratory viruses can be attributed to a combination of factors, including the survival of the virus in different environmental conditions, changes in contact patterns (such as school holidays), less time spent in closed spaces where the highest number of transmissive contacts are made, and potentially seasonal changes in the health conditions of the population as well. To express this behavior, we define a time-dependent parameter

(3) $$\omega \left(c,t\right)=0.5\cdot c\cdot \cos\left(\frac{2\pi t}{366}\right)+\left(1-0.5\cdot c\right)$$

by which we scale the transmission rate β. Parameter *c* denotes the magnitude of the effect of seasonality on the number of contacts. Using such a time-dependent transmission rate, we compare possible disease dynamics generated by the interplay of control measures with different degrees of seasonal behavior.

Spatial heterogeneity is also considered using our patch model, where the country is divided into distinct geographic regions (patches). The transmission dynamics is described within each patch by our compartmental model (but potentially with different parameters and age group composition), and individuals may move between those patches. For obvious reasons, individuals in compartments $I_{h}^{i},I_{c}^{i},I_{\mathrm{cr}}^{i}$ and $D_{}^{i}$ do not travel. Let ${\mathrm{travel}}_{p,q}^{}$ denote the number of travels from patch *p* to patch *q*. To derive travel rates $t_{p,q}^{}$ for each age group *i*, we divide the number of travels with the population of the appropriate patch

$$t_{p,q}^{i}=\frac{{\mathrm{travel}}_{p,q}^{}}{N_{p}^{}\left(t\right)}.$$

Numerical simulations for such situations show the differences in the transmission dynamics, healthcare demand, mortality, and overall disease burden. These scenarios are summarized in Table 5.

#### 2.3.6. Parameter Uncertainty and Other Limitations

Our work has several limitations. Due to limited testing and the large number of asymptomatic and mild cases, there was a huge uncertainty in the number of true cases, especially in the early weeks. Now, with the help of [58], we have a good estimation of the overall ascertainment rate over this period, but it is still unclear how this rate evolved in time. The transmission model has the same weaknesses that all compartmental models have: we assume a homogeneous population with random mixing, apart from the age structure. We added some further heterogeneity in space (patch model) and time (seasonality). In our scenarios, we assumed a constant reduction in transmission, while in reality the control measures and the behavior of the people were continuously changing. Hence, such scenarios cannot be considered as predictions, as we cannot expect such unchanging circumstances for months. The role of children in this pandemic is still not clear, in our modeling, we assumed that they are equally susceptible, and equally infectious once they develop symptoms, but we used an age-specific probability for developing symptoms.

Since our transmission model is deterministic, it is suitable only when there is significant spread in the population. For very low case numbers, the development of the epidemics is largely influenced by random events. Stochastic effects are important when considering extinction or resurgence of the disease, and possible case importations after travel restrictions are lifted. However, these issues are not in the scope of the present work.

The model has a large number of parameters, many of those have uncertainty. The most important ones in regard to the burden on the healthcare system are hospitalization rates, probability of intensive care need, mortality, all of those depending on age. We do not have too much data for this from Hungary, hence we used parameters taken from the literature. A full sensitivity analysis is beyond the scope of this study, but we present a sensitivity chart for a crucial output of an outbreak, see Section 3.8, which is of concern in many countries: the peak ICU demand, including the need for mechanical ventilators, to assure that all patients receive the necessary care, and no additional excess mortality is caused by an overwhelmed healthcare system. This was one of the key questions in other modeling studies. The sensitivity analysis was conducted by running many simulations, sweeping through a two-parameter plane, and retrieving the ICU peak from each individual run. The code can be found in [49].

## 3. Results

### 3.1. Epidemiological Report

**Figure 3:**
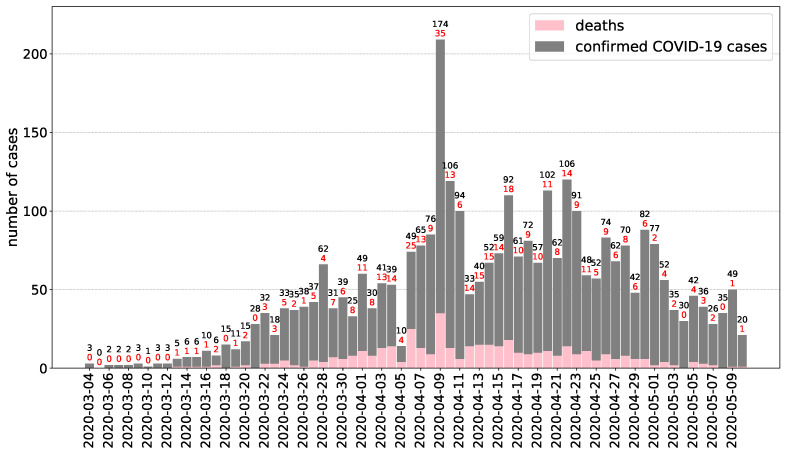
Epidemic curve of confirmed COVID-19 cases in Hungary by date of confirmation (reported until May 10, 2020. Data source: NPHC).

The first Hungarian COVID-19 cases were reported during the first week of March 2020 through the Hungarian Notifiable Disease Surveillance System operated by NPHC which is the source of data described in this section (for the most recent information, see [10]). The first case, an Iranian 27-year old man (studying and residing in Hungary) who recently returned from Tehran, was reported on 4 March 2020. By 10 May 2020, the cumulative number of reported confirmed COVID-19 cases were 3284 (33.1 cases per 100,000 population), including 421 deaths (crude CFR 12.8%), see Figure 3 for the daily reported numbers.

Out of the 3284 cases, 47.0 % (1,542 cases) occurred in the 65+ age group, 29.1% (957 cases) in the 20–49 age group, 21.9% (718 cases) in the 50-64 age group and 2.0% (67 cases) among people under 20-years old. Age specific morbidity was highest in the 80+ age group (163.3 cases per 100,000 population) and more than twice of the overall in the 70–79 age group (69.5 cases per 100,000 population).

**Figure 4:**
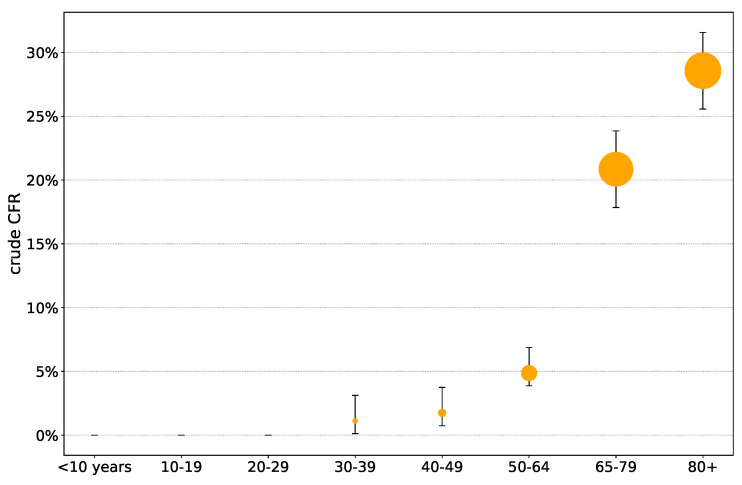
Crude case fatality rate of confirmed COVID-19 cases in Hungary by age groups (reported until May 10, 2020. Data source: NPHC).

Out of 421 deaths, 89.1% (375 deaths) belonged to the 65+ age group. As seen in Figure 4, the highest crude CFR was observed in the 80+ age group (28.6%), followed by the 70–79 age group (22.4%) and the 65–69 age group (17.3%). No deaths were reported under 33 years of age, see Figure 4. Additional details are provided in Table 6.

Out of the 3284 cases, 58.0% (1906 cases) were female and 41.9% (1376 cases) male (gender is unknown for two cases). The morbidity among women was higher (37.1 vs. 28.8 cases per 100,000 population), so men were 0.8 (95% CI 0.72–0.83) less likely to become ill. However, men aged 33 years and older had a 1.2 (95% CI 0.96–1.41) higher risk to die than women aged 33 years and older (7.1 cases vs. 6.0 cases per 100,000 population). Out of 3284 cases, at the stage of data consolidation as of May 10, 2020, we have information about the symptoms of 63.2% (2076 cases). Out of 2076 cases, 29.5% (613 cases) had no symptoms, 52.3% (1086 cases) had mild symptoms, and 18.2% (377 cases) had severe disease (including 149 cases required intensive care and/or ventilation).

**Figure 5:**
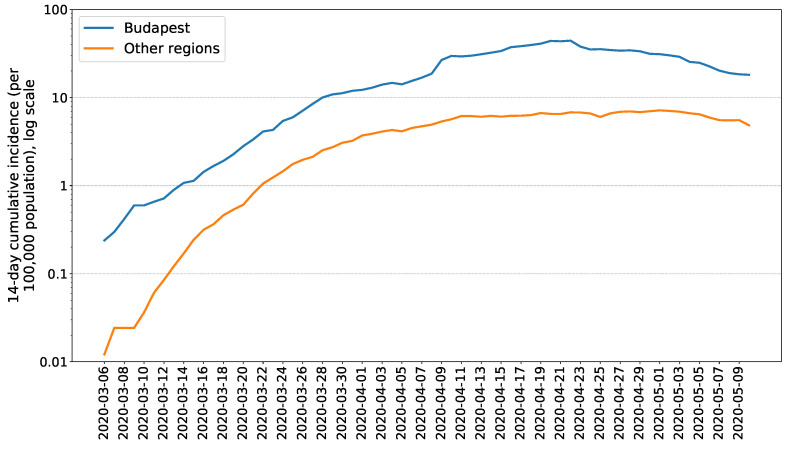
Fourteen-day cumulative incidence of confirmed COVID-19 cases in Budapest and in other regions of Hungary (reported until 10 May 2020. Data source: NPHC).

Most of the cases were reported from the central part of Hungary, from the capital (1587 cases) and the surrounding Pest county (438 cases). See Figure 5 for a comparison of the capital region with the rest of Hungary. The morbidity (per 100,000 population) was also the highest in Budapest (94.7).

The epidemic curve (Figure 3) reflects a propagated source epidemic especially when we consider only those cases that cannot be connected to outbreaks in closed communities (like long-term care facilities or hospitals) or to health care associated infections. Out of 3284 cases, 31.4% (1031 cases) were associated with health care and/or outbreaks in hospitals, contributing to the daily reported new cases since mid-March. Health care workers had 10.0 times (95% CI 9.02–10.99) higher risk to become a confirmed COVID-19 case in comparison to the general population (288.8 cases vs. 29.0 cases per 100,000 population). Out of 3284 cases, 27.8% (913 cases) were reported from long-term care facilities (nursing homes and other closed communities like homeless shelters) contributing to the daily reported new cases since early April. At the peak of the epidemic curve, 62.2% (130 cases) of cases on April 9 were reported from the same retirement and assisted living facility.

### 3.2. Statistical Analysis

**Figure 6:**
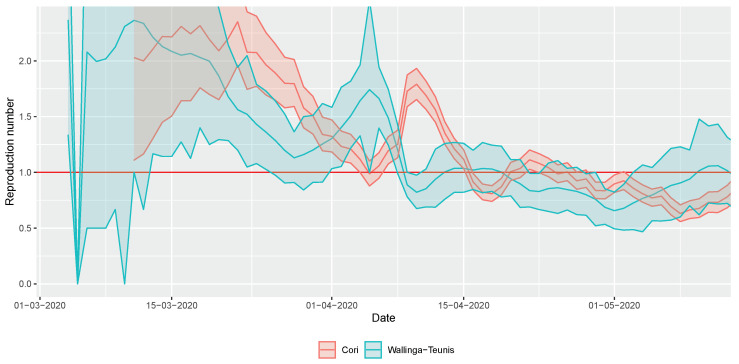
Real-time estimation of the reproduction number during the early phase of the COVID-19 outbreak in Hungary using two different methods (shaded area depicts 95% confidence interval).

Figure 6 shows the results for the real-time estimation of the reproduction number. It showed a steady decline—apart from an outlying effect in early April—and became close to, or even below 1 by mid-April, and remained at that level since then. This conclusion is robust to the chosen methodology.

**Figure 7:**
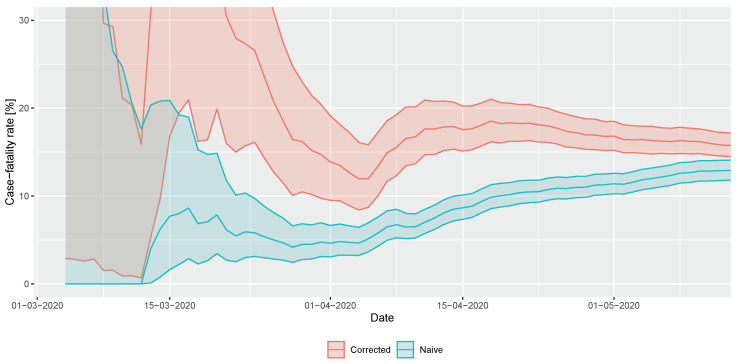
Real-time estimation of case fatality rate during the early phase of the COVID-19 outbreak in Hungary (shaded area depicts 95% confidence interval).

Results for the real-time estimation of CFR are shown in Figure 7. Note that—as the outbreak is coming to its end—the naive method converges to the final value that was readily well estimated almost a month earlier by the corrected technique. (The naive estimator is increasing as deaths still occur, but case count is already low at the end of the epidemic.) The final CFR to characterize this phase in Hungary is about 16%.

Various IFR estimations have been published, for example 0.66% for China [59], 0.9% for UK [41]. Recent serological studies found IFR values spanning from 0.36% in a German town [60], to 1.19% in Milan [61]. Note that the testing intensity—and therefore the ascertainment rate—may very well change over time, e.g., with the increase of testing intensity. This analysis is based on the data from the early phase as a whole and, therefore, it is considered as an estimation of the average.

The results for the estimation of the ascertainment rate are shown in Table 7, where we explore a reasonable range of IFRs from 0.3% to 1.2%. Note that earlier estimates based on [41,59] are consistent with the preliminary results of a large-scale Hungarian sero-epidemiological study [58].

### 3.3. Post-Lockdown Scenarios

Most studies concerning the early growth-rate of the epidemic in Wuhan estimated the value of the basic reproduction number to be around 2.0–3.0 (see e.g., [45,62]), also later studies regarding the spread in other countries [41,42] used similar values. Our estimations given in Section 2.2 shows that in Hungary the highest value of the effective reproduction number was 2.2, by the Wallinga–Teunis method. Hence, we choose $R_{0}=2.2$ for the basic reproduction number (comparable with a similar reproduction number for Germany in the early phase [63], 3.2 for Italy [64]). Modeling studies [38,39,41,42] highlighted that the worst case, i.e., “do nothing” scenarios lead to an outbreak when the healthcare demand substantially exceeds the capacities at the peak and the overall mortality reaches severe levels. Given the current level of preparedness, we do not consider a “do nothing” scenario, and our most pessimistic case assumes that, even in the absence of any control measures, a 25% reduction in transmission is realized due to population awareness and behavior.

On the other hand, the best case is the continuation of the current suppression scenario with R≈1, resulting in very small case numbers. However, it is questionable whether it can be sustained until a vaccine is developed and deployed. Below, we consider three scenarios illustrating the loss of control for suppressing the outbreak, and assuming a wide community spread of the disease. The efficacy of the mitigation efforts is expressed by a percentage in the reduction of transmission. The primary tool for this is the decrease of contact numbers, but other preventive measures such as hand hygiene or mask wearing may also have an effect in the reduction of transmission.

**Figure 8:**
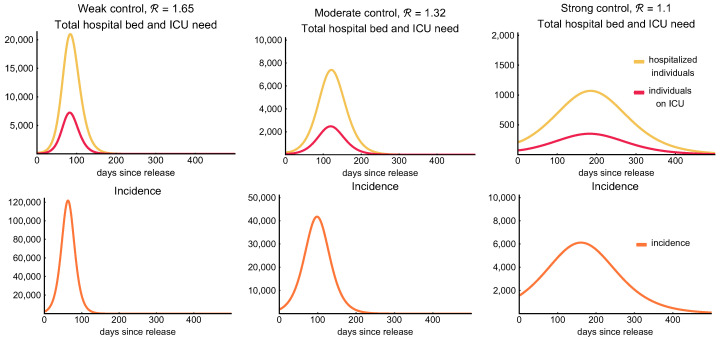
Hospitalization, ICU demand, and incidence curves. The figures show the required number of hospital beds (classes $I_{h}+I_{cr}$, yellow) and ICU beds (class $I_{c}$, red) need in the first row for R∈{1.65,1.32,1.1}, respectively. The second row illustrates the daily incidence (transition from compartment *S* to $L_{1}$ in our model, orange) combining all age groups. Note that the incidence curves peak earlier than the hospitalization curves. The legend at the bottom applies for all figures. Note that the scalings of the figures are different.

First, let us consider a weak control of the epidemic assuming there is no centralized control measure introduced, but the number of transmissions is reduced by 25% following a level of behavioral response due to social awareness. Such a reduction decreases the reproduction number to R=1.65. The first column of Figure 8 shows the hospitalization and ICU demand on the top row and the daily incidences on the bottom row as a function of time with the application of this weak control. According to the simulations, in this case, there would be approximately 5.7 million infections with about 22,000 deaths by the end of the outbreak. This suggests that we can expect 58% of the population to gain immunity against the virus and this number is slightly larger than the threshold of herd immunity (that is $(1-1/R_{0})∼54.5\%$ with $R_{0}=2.2$ for the “do nothing” scenario). At the peak, there would be a need for more than 7200 ICU beds and for 21,000 hospital beds with such a weak measure. We remark that there is a 20-days window when the daily incidences exceed 100,000, and during this period more than 2.64 million people (27% of the population) get infected. In other words, 40% of all the infections occur during these three weeks. For further details, see Table 8.

We perform similar simulations for the case of a moderate control, assuming that the reproduction number is decreased to R=1.32 as a result of the control measures. The simulations (second column of Figure 8) show that the number of hospital beds and ICU beds needed is significantly reduced to 7400 and 2500 at the peak, respectively. Meanwhile, the daily incidence at the peak is around 40,000. We expect almost 37.5% percent of the population to be infected throughout the epidemic and gain immunity upon recovery. This is less than required to reach herd immunity. For further information, we refer to Table 8.

Finally, we consider a stronger control achieving a 50% reduction of transmission. This results a decrease of the reproduction number to R=1.1. The outcome of this strong control is shown in the third column of Figure 8. A control of such strength significantly reduces the number of all infected and hospitalized cases and of those needing intensive care treatment. The number of required intensive care beds (around 350) is far below the available capacity even at the peak of the epidemic and also the number of hospital beds needed is reduced to a rather low level—around 1100 at the peak. The total number of fatalities in this scenario is about 4500. Meanwhile, the epidemic would last for more than a year and the cumulative number of all infected remain far below the level of herd immunity threshold, so we can expect further outbreaks when the measures are relaxed.

### 3.4. Age-Dependent Intervention Measures

Several key parameters of the model are highly dependent on age. Intervention strategies and the relaxation of various measures have to take into account the fact that different age groups have different risks and different roles in the transmission.

Although the number of children infected with COVID-19 has been reported worldwide relatively small in comparison with other age groups [65], some evidence shows that children and adolescents may become infected and spread the disease as other age groups [66,67]. Moreover, children and adolescents usually have a high number of contacts. Thus, school closures can be expected to be an efficient tool to reduce the contacts and transmissions. Besides school closures, it is important for younger individuals to avoid meeting older and other high risk people.

Elderly people have a higher chance of developing symptoms, and a higher percentage of them needs hospitalization and intensive care, hence these groups need more protection. Age-specific interventions include avoiding contacts with elderly by providing special time slots for shopping, in post offices, etc., or closing/reopening schools.

**Figure 9:**
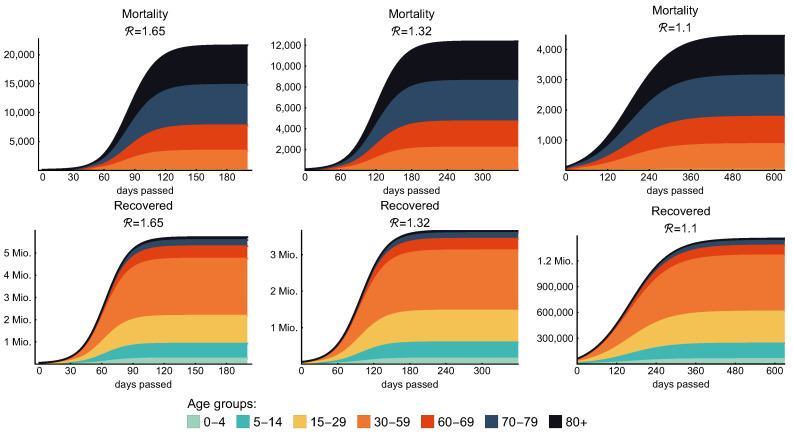
Age-specific mortality and recovery. The figure shows the effect of the weak, moderate, and strong control (25%, 40% and 50% general contact reduction, respectively). Every age group covers at most one decade except the group of “middle aged” that represents three decades. According to our model, elderly people (60+) are predicted to produce most of the fatality cases in each scenario. The legend on the bottom applies for all figures.

The introduction of various age groups in our model enables us to study such age-specific interventions and analyze their direct and indirect effects on all groups. On the stacked diagrams of Figure 9, we present the contributions of the age groups to the mortality and the number of recovered individuals. Columns of this figure show the effect of the weak, moderate, and strong control that we previously discussed in details in Section 3.3 and Table 8. Here, we would like to emphasize that, in the case of each control measure, the most vulnerable age groups are the groups of elderly (60–69, 70–79, 80+) people as they suffer most of the fatalities; meanwhile, they are predicted to produce only a small fraction of the cases in the population.

#### 3.4.1. School Closures

We consider two school closure scenarios: an optimistic and a pessimistic one (with respect to the outcome of the outbreak); both use the weak control scenario (25% general decrease in transmission, cf. Section 3.3) as a starting point. The optimistic case is comprised of omitting the school component of the contact matrix and halving the *other* contacts [56] of children and young adults (between age 0–29), which provides a new global contact matrix for this intervention. In the pessimistic scenario, we omit the school component of the contact matrix as well, but, instead of halving, it considers a 25% increase in the *other* contacts of children and young adults. Arguably, the students might replace some school contacts by new *other* contacts, due to other activities. However, many of such contacts are lost as well: for example, they do not use public transportation to/from the school, and extracurricular activities also drop. Since the exact balance is difficult to estimate, our two closure scenarios serve as a boundary to explore this regime of possibilities. Note that, by school closure, we mean the closure of educational institutions from preschools to universities. As a reference, we also incorporate the weak control scenario to this analysis.

**Figure 10:**
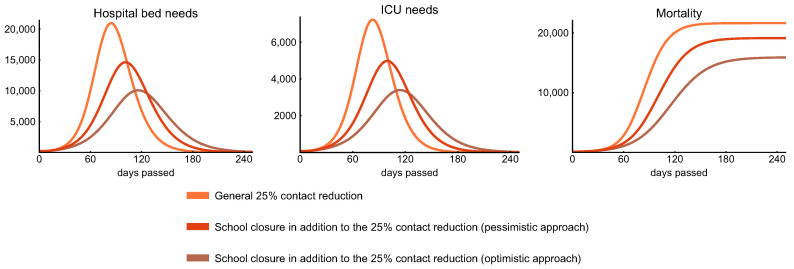
Effect of school closure. Simulations suggest that school closures—if maintained for a long period—effectively decrease peak hospital bed and ICU needs and significantly postpone the peak of the epidemic.

Figure 10 shows that this measure decreases the peak hospital bed and ICU needs to approximately 50% compared to the case when we only apply weak control in the optimistic scenario and by 25% in the pessimistic one. Moreover, closing schools postpones the peak of the epidemic (by about one month in case of the above setting), suggesting that children may play a significant role in transmission due to their large number of contacts, even though they give negligible contribution to the overall mortality, cf. top row of Figure 9). Note that this conclusion is based on the assumption that all age groups are equally susceptible, and symptomatic children are equally infectious to adults, and age specific difference appears only in the probability of developing symptoms, which is much smaller for children in our model (see parameters $p^{i}$ in Table 3).

The effect of school closure combined with the 25% general reduction in transmission is comparable, in the optimistic case, with the effect of moderate control (40% reduction in transmission, cf. Section 3.3) regarding the peak hospital bed and ICU need, but not as significant in decreasing the mortality (Figure 9 middle column). However, to achieve this, schools need to be closed for an extended period of time, which may not be feasible. We also point out that a standalone closure of preschools and primary schools is not sustainable without a certain amount of home office of the parents, but this opens up sociological and economical questions that we do not address here.

#### 3.4.2. Protection of the Elderly

The elderly being the most vulnerable group of the population, when it comes to relaxation of measures introduced against the spread of COVID-19. Most countries handle these age groups separately from the rest of the population, e.g., separate time slots for shopping continue to exist and elderly are encouraged to keep the same level of social distancing [10,68]. To include these effects in our model, we manipulate the entries of the contact matrix involving older age groups separately from the remaining parts.

**Figure 11:**
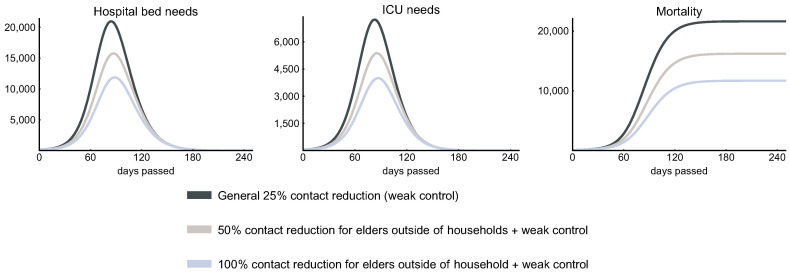
Protection of the elderly. The figures show the effect of an additional contact reduction of elderly people in case of a weak control. The figures suggest that the selective protection of elderly people can successfully reduce the peak ICU need and the overall mortality, yet it has a theoretical limit.

Figure 11 illustrates that, in addition to the weak control, if 50% and 100% reduction of the outside household connections of elderly people is applied, then we can expect about 25% and 50% reduction in the hospital, ICU bed needs, and mortality. The epidemic curves only slightly shift to the right suggesting that elderly people do not play an important role in the transmission of the disease due to their low number of contacts. In addition, 100% reduction of contacts outside the household is again not feasible, as this would mean the complete isolation of a large sub-population. We plotted this scenario only to show the theoretical limits of this approach.

### 3.5. Role of Seasonality

**Figure 12:**
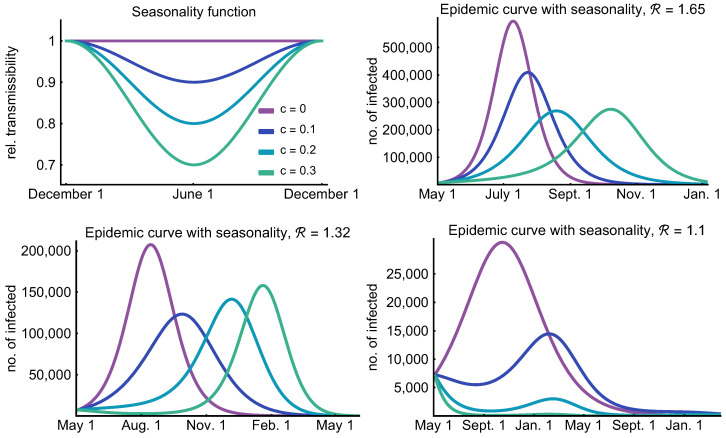
Effect of seasonality. The top left figure depicts the relative transmissibility of the virus throughout the year. Purple denotes no seasonality (c=0), blue curves correspond to weak seasonality (c=0.1), turquoise curves stands for moderate seasonality (c=0.2), and green curves correspond to strong seasonality (c=0.3). The next three figures investigate the number of infected individuals under these seasonality scenarios with R=1.65,R=1.32, and R=1.1, respectively.

In this section, we investigate the epidemic curves in case of the weak, moderate, and strong control with seasonality of various strengths expressed by parameter c∈{0,0.1,0.2,0.3}, see (3). During the summer, these values of *c* eventuate a 10%, 20%, and 30% further decrease in transmission as that is when the seasonality curve attains its minimum. The case c=0 means that there are no seasonal effects at all, while c=0.3 is a strong seasonality, which is similar to H1N1 [69]. See the top left image of Figure 12 for the seasonality functions ω(c,t) corresponding to the different *c* values.

As we have seen in Section 3.3, decreasing the reproduction number decreases and postpones the peak of the epidemic curves. Seasonality causes a similar delay in the peak of the epidemic due to decreased transmission rates in the summer months. Counter-intuitively, it cannot be said in general that stronger seasonality leads to a smaller peak (cf. bottom left image of Figure 12). The reason for this is that the impact of seasonality is not only determined by the decrease in the transmission rate, but the temporal relation between the peak of the epidemic and the minimum of the seasonality function is also an important factor. This phenomenon is well illustrated in Figure 12 where three scenarios (weak, moderate, and strong control) are presented along with the assumed seasonality functions for the aforementioned values of *c*.

In the upper right image of Figure 12, corresponding to a weak control, one can observe that increasing the effect of seasonality first decreases the peak, but, after a certain value (c=0.3 in our example), the epidemic is so much suppressed in the summer months that the peak shifts to the right and even slightly increases in winter months compared to the c=0.2 scenario.

For the case of moderate control, shown in the lower left figure, this effect is much more significant. Note that the peak of the epidemic (without seasonality) is so far from summer (the minimum of the seasonality curves) that increasing the effect of seasonality results in a significantly higher peak. It can be seen that strong seasonality eventuates a long “plateau” phase when the epidemic curve does not increase in a period of six months. During this time, only a small fraction of the population goes through the infection and a massive number of susceptibles remain in the system, only to get infected a few months later. This phenomenon is responsible for the increased peak of c=0.3 compared to the c=0.2 case.

The lower right figure shows that the reduction of transmission during the warm months together with a strong control can decrease the number of infected in such an extent that the peak, even if arriving in the winter months, is significantly smaller.

A general observation is that seasonality has the largest impact on the epidemic curve if the peak time is close to the summer months. Of course, this is highly dependent on the starting time of the outbreak.

### 3.6. Spatial Heterogeneity

Hungary is a relatively small country; however, significant differences were observed between regions in the reported case numbers. The capital, Budapest, has 1.75 million inhabitants and a further 1.23 million people live in its surrounding Pest county. Budapest and Pest county are highly connected by commuters with connections to other regions as well [70]. The high connectivity of the capital with other countries contributed to the earlier appearance of the disease in Budapest, and most of the cases were reported from this central region of the country.

To address the role of spatial heterogeneity in the evolution of the epidemic curve, we considered a metapopulation model as in (2). Hence, the population is distributed among patches, representing geographic regions of the country. For the sake of simplicity, here we only present results from a two-patch model, separating Budapest and Pest county (patch 1, population of approx. 3,111,000) from the remaining parts of the country (patch 2, population 6,610,000).

We assumed different transmission parameter $\beta_{}^{}$ for each patch. Based on Hungarian mobility data on commuters [70], we assumed 400,000 daily travels between the two patches in the case of normal circumstances and investigated the effect of the lockdown of Budapest and the surrounding Pest county by decreasing the number of daily travels to 10,000. We considered the contact matrix for both patches to be the same as in the uniform model described in Section 2.3.3. The biological and medical parameters are assumed to be the same in each patch, but the local reproduction number may differ, as well as the age structure of the population.

**Figure 13:**
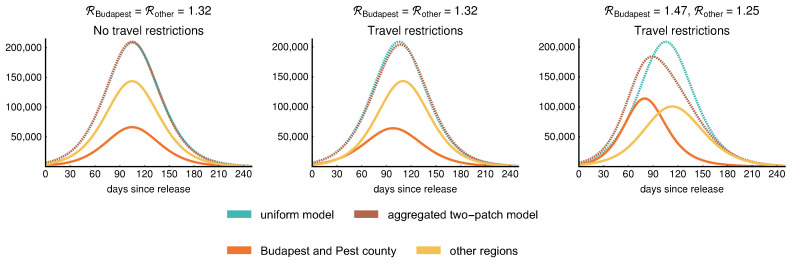
Epidemic curves of the regions: sum of the infective compartments $\left(I_{p},I_{a,1},I_{a,2},I_{a,3},I_{s,1},I_{s,2},I_{s,3}\right)$. First, we consider identical reproduction numbers R=1.32 for both patches (Budapest with Pest county and other regions). Without any travel reductions, the two-patch model gives identical results to the one-patch version, as seen in the left figure. Next, if travel reductions are put in place, the one-patch model overestimates slightly the size of the epidemic for equal R values. Finally, assuming different reproduction numbers and large reduction in travel, the peak occurs earlier in the patch with larger R (Budapest and Pest county); furthermore, the one-patch model and the aggregated two-patch model differ in both time and size of the peak.

The left-hand side of Figure 13 illustrates that the two-patch model reproduces the uniform model in case we use the same R=1.32 for both patches as well as for the uniform model and we assume 400,000 daily travels between the patches. The middle figure shows that the uniform model slightly overestimates the size of the epidemic as the peak of the aggregated two-patch model is smaller than that of the uniform model in case R=1.32 remains the same, and we reduce the daily travels to 10,000 corresponding to the separation of Budapest and Pest county from other regions. Although the epidemic curves of the patches are shifted, the aggregated result shows that this setup does not provide significantly different dynamics. Lastly, on the right-hand side of Figure 13, we further investigate the scenario of 10,000 daily travels, and choose the local reproduction numbers of the patches to vary around R=1.32, namely, we take $R_{\mathrm{Budapest}}=1.47$ and $R_{\mathrm{other}\mathrm{regions}}=1.25$. These values were selected to reflect the higher population density of the capital, proportionally to the population in the two patches. Due to the difference in the local reproduction numbers, we may observe an increased number of cases in Budapest with an earlier peak and fewer infections in other regions.

### 3.7. Sensitivity of the Peak ICU Demand to Key Parameters

For an uncontrolled epidemic in the UK, Ref. [41] estimated a peak in ICU bed demand more than 30 times greater than the maximum capacity in these countries. In a study for the United States, Ref. [42] projected that, at the outbreak peak, three times more ICU beds would be needed than the total number of ICU beds in the US, and 85% isolation of cases reduces the demand for ICU beds to the normal capacity. In the Île-de-France region, Ref. [39] estimated that the peak number of ICU beds needed would exceed more than 40 times the regional capacity if no strategy is implemented after lockdown, and only efficient case-finding and isolation applied parallel with social distancing could decrease ICU demand below the maximum capacity throughout the epidemic. For Australia, Ref. [38] studied three capacity expansion scenarios (2, 3 and 5 times expansion, respectively), and, even in mitigated scenarios, demand is estimated to be higher than the number of available beds. Additional social distancing measures were shown to reduce the epidemic to a level where a reasonable expansion of ICU capacity can be sufficient.

**Figure 14:**
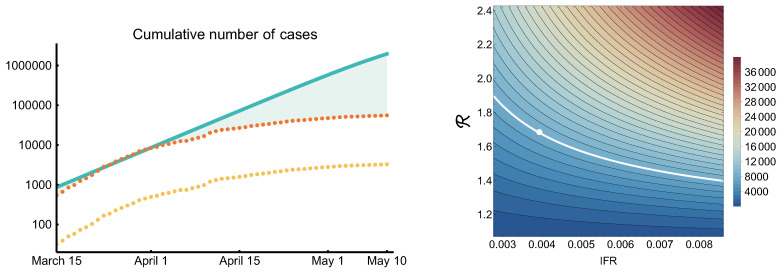
Left: the impact of control measures on the epidemic trajectory. Yellow dots are cumulative numbers of reported cases, orange dots are corrected data by underascertainment rate, solid curve is simulated cumulative numbers with R=2.2 and the absence of measures. Right: Sensitivity of the peak ICU demand to transmissibility and severity of COVID-19. The top right corner is similar to the worst case scenario of [41]. The white dot is our most pessimistic scenario (weak control).

The peak ICU demand crucially depends on two factors: the probabilities of developing severe disease, and the shape (in particular the peak size) of the epidemic curve. We plotted a heatmap of the peak ICU demand in Figure 14, compiled from hundreds of numerical simulations. Transmissibility (vertical axis) is expressed by the reproduction number R. Disease severity, for simplicity, is expressed by the IFR. In fact, here we used a scaling factor for the probability of hospitalization, with the baseline corresponding to the parameters in Table 3. In our weak control scenario (Section 3.3), the IFR is 0.4%, which is a bit lower than the finding of [58]. However, during the first wave in Hungary, the schools were closed and COVID-19 disproportionately affected the vulnerable population. In our scenarios, we assume a widespread community spreading, hence younger generations appear in higher numbers, thus the IFR is expected to be smaller. In any case, by the scaling of the hospitalization rate (while leaving the probability of intensive care and fatal outcome given hospitalization intact), we explored a wider range of IFRs. We found that indeed the peak ICU demand can vary across a large interval. From the shape of the level curves in the heatmap, we can conclude that the peak ICU demand is more sensitive to R than to the IFR, hence flattening the curve is indeed of utmost importance to avoid exceeding healthcare capacities.

### 3.8. The Impact of Implemented Measures Since Mid-March

The most important implemented measures are summarized in Table 9. To assess their impact, we compared the reported case numbers adjusted by the ascertainment rate 1:17 to the simulated outbreak curve with R=2.2 (Figure 14 on the left, logarithmic scale). Here, we assumed that the ascertainment rate did not change in time, which may not be the case. One can see that the epidemic was on the R=2.2 trajectory, which could have resulted in substantially more infections. The data shows a clear deviation from this scenario early April, two weeks after strict social distancing started. The slope of the epidemic curve further decreased mid-April, following the stay at home measures by two weeks.

Overall, due to the compliance of Hungarian society with the social distancing measures, around half million infections were averted by the end of April, compared to the “do nothing” scenario, which could have reached 1-2 million in May if further doublings would have been allowed.

## 4. Conclusions

The first COVID-19 case was detected, the laboratory confirmed, and then reported through the Hungarian Notifiable Disease Surveillance System on 4 March 2020. Well tailored, effective, combined non-pharmaceutical control measures have been introduced promptly in Hungary in the very early phase of the outbreak (see Table 9), accompanied with a high level of compliance for social distancing. Online surveys [48], polling, and indirect data (such as traffic data, passenger volumes on public transportation, etc.) all showed a drastic reduction in the number of contacts and mobility. In particular, the online questionnaire MASZK [48] showed a 60–90% decrease (depending on the locality) in the daily number of physical contacts as well as in the number of closed contacts per capita, based on the replies of 380,000 respondents by May 10, constituting a non-representative, but rather large sample. Accordingly, the Hungarian epidemic curve was strongly suppressed. As of 10 May 2020, the cumulative number of reported confirmed COVID-19 cases were 3284 (33.1 cases per 100,000 population), including 421 deaths. The epidemic peaked on April 9 with 209 newly reported cases. SARS-CoV-2 was not able to sustain long transmission chains in the community; however, it was able to cause outbreaks mostly in healthcare institutions and long-term care facilities: nearly two thirds of the reported cases are connected to such institutions. The proportion of cases in health care workers gradually increased during the epidemic. They had tenfold risk to become confirmed COVID-19 cases compared to the general population. Due to effective measures, the virus could not spread significantly from closed communities and health care workers to the wider population. The age specific CFR showed a similar pattern to other countries: of the 421 deaths reported by 10 May, 375 (89.1%) belonged to the 65+ age group.

We tracked the temporal variation of the effective reproduction number in real time, which showed a steadily decreasing trend, interrupted by an outlying outbreak in a long-term care facility. We identified the time intervals when the effective reproduction number was below or around the critical threshold 1. The adjusted CFR was also estimated real-time, and predicted the eventual CFR one month in advance well. Benchmarking the CFR to other countries, we estimated underascertainment rate to be 10–20 times, and the true cumulative number of COVID-19 cases to be between 32,840 and 65,680. These results are consistent with data from the preliminary results of a large scale seroepidemiological survey, carried out in Hungary in May 2020, where the seroprevalence of SARS-CoV-2 infection was estimated to be between 22,399 and 92,624 [58]. Based on these data and the number of reported cases, underascertainment is likely to be between 6.8–28.2, and the true CFR may be lower than 1.5%, and the IFR is roughly half of that.

As control measures are being successively relaxed since May 4, we established an age-structured compartmental model to investigate several post-lockdown scenarios, and projected the epidemic curves and the demand for critical care beds assuming various levels of sustained reduction in transmission. Special measures designed to reduce the contact number of the elderly population as well as school closures can reduce the peak hospital bed demand and the overall mortality; however, these measures also have their limitations. A metapopulation version of the transmission dynamics model has also been studied, and we reported some results for a two-patch case, where the Budapest region is considered separately from the rest of the country. Due to the high connectedness, the epidemic curves of the two-patch system are not much different from the spatially uniform case. To achieve a noticeable reduction in the overall peak size due to spatial heterogeneity (where the local peak times are shifted in the regions), a large reduction in the mobility rates is necessary.

Since the majority of the population is still susceptible (over 99%, according to [58]), a weak or even a moderate reduction in the transmission, compared to the baseline, could result in a large second outbreak with significant mortality and high peak ICU demand. Therefore, a high level of alertness needs to be maintained to avoid such scenarios.

The seasonal behavior of SARS-CoV-2 is not completely understood yet [71,72], thus we considered a range of possibilities from the absence of seasonality to a strong seasonality, which is similar to H1N1. The interplay of seasonal effects with the post-lockdown contact numbers can generate a variety of disease dynamics; thus, a confident forecast of the timing and the size of a potential second wave is not possible at the moment.

The effectiveness of strict social distancing measures, such as school closures and stay at home measures with good compliance is likely to be very high; however, such interventions have negative consequences on the society and on the economy and are thus not sustainable in the long term. Modeling results [73,74] suggest that combined multiple interventions, including moderate contact decrease, high COVID-19 detection rate, effective contact tracing, and good compliance with personal protective instructions, may have substantial impact on transmission, and are able to keep the reproduction number around one.

## Figures and Tables

**Table 1:** Age-independent epidemiological parameters of COVID-19. Assumed to be valid for all age groups. References and explanations are in Section 2.3.2.

Duration of		Value
Incubation period	${\left(\alpha_{L,1}^{i}\right)}^{-1}+{\left(\alpha_{L,2}^{i}\right)}^{-1}+{\left(\alpha_{p}^{i}\right)}^{-1}$	5.2 days
Latent period	${\left(\alpha_{L,1}^{i}\right)}^{-1}+{\left(\alpha_{L,2}^{i}\right)}^{-1}$	3.2 days
Presymptomatic (infectious) period	${\left(\alpha_{p}^{i}\right)}^{-1}$	2.0 days
Infectious period of $I_{a}^{i}$	${\left(\gamma_{a,1}^{i}\right)}^{-1}+{\left(\gamma_{a,2}^{i}\right)}^{-1}+{\left(\gamma_{a,3}^{i}\right)}^{-1}$	3.0 days
Infectious period of $I_{s}^{i}$	${\left(\gamma_{p,1}^{i}\right)}^{-1}+{\left(\gamma_{p,2}^{i}\right)}^{-1}+{\left(\gamma_{p,3}^{i}\right)}^{-1}$	3.0 days
Hospitalization	${\left(\gamma_{h}^{i}\right)}^{-1}$	10.0 days
Intensive care		
until transition to $R_{}^{}$ or $I_{\mathrm{cr}}^{i}$	${\left(\gamma_{c}^{i}\right)}^{-1}$	10.0 days
Recovery in $I_{\mathrm{cr}}^{i}$	${\left(\gamma_{\mathrm{cr}}^{i}\right)}^{-1}$	14.0 days
**Relative infectiousness**		
Presymptomatic vs Symptomatic	$\beta_{p}^{\left(k,i\right)}/\beta_{s,\_}^{\left(k,i\right)}$	1.0
Asymptomatic vs Symptomatic	$\beta_{a,\_}^{\left(k,i\right)}/\beta_{s,\_}^{\left(k,i\right)}$	0.5

**Table 2:** Age groups of the Hungarian population.

Age Group	0–4	5–14	15–29	30–59	60–69	70–79	80–
**Population**	468,605	953,134	1,678,211	4,087,976	1,312,208	839,589	433,033

**Table 3:** Age-dependent epidemiological parameters of COVID-19.

Probability/Age Group		0–4	5–14	15–29	30–59	60–69	70–79	80–
Asymptomatic course	$p_{}^{i}$	0.95	0.8	0.7	0.5	0.4	0.3	0.2
Hospitalization or								
intensive care (from $I_{s,3}^{i}$)	$h_{}^{i}$	0.00045	0.00045	0.0042	0.0442	0.1162	0.2682	0.4945
Intensive care								
(given hospitalization)	$\xi_{}^{i}$	0.333	0.333	0.297	0.294	0.292	0.293	0.293
Fatal outcome								
(from $I_{\mathrm{cr}}^{i}$)	$\mu_{}^{i}$	0.2	0.2	0.216	0.3	0.582	0.678	0.687

**Table 4:** Baseline transmission rates from NGM computation.

R	1.0	1.1	1.32	1.65	2.2
**β**	0.0210	0.0231	0.0277	0.0347	0.0462

**Table 5:** Scenarios.

Scenario	Description	Pointer
Weak control	general 25% reduction in transmission	Section 3.3
Moderate control	general 40% reduction in transmission	
Strong control	general 50% reduction in transmission	
School closure	two variants of changing the mixing patterns of schoolchildren	Section 3.4.1
Protection of elderly	50–100% reduction of contacts outside the household for the elderly	Section 3.4.2
Seasonality	exploring various degrees of seasonal behavior	Section 3.5
Spatial heterogeneity	considering two patches, which are strongly or weakly connected	Section 3.6

**Table 6:** Number of confirmed COVID-19 cases and deaths, morbidity (per 100,000 population) and crude CFR of confirmed COVID-19 cases in Hungary by age groups.

Age Group (Years)	Number of Confirmed COVID-19 Cases	Morbidity (Per 100,000 Population)	Number of Deaths	Case Fatality Rate (Per 100 Confirmed COVID-19 Cases)
<1	3	3.3	0	0.0
1–4	10	2.6	0	0.0
5–14	31	3.2	0	0.0
15–29	254	15.5	0	0.0
30–39	267	19.9	3	1.1
40–49	459	27.7	8	1.7
50–59	485	38.3	15	3.1
60–64	233	35.4	20	8.6
65–69	254	39.4	44	17.3
70–79	595	69.5	133	22.4
≥80	693	163.3	198	28.6
Overall	3284	33.1	421	12.8

**Table 7:** Underascertainment (ratio of all infections to reported cases) and corrected number of cumulative cases based on the estimated underascertainment.

IFR	0.3%	0.6%	0.9%	1.2%
Underascertainment (true/reported)	54.0	27.0	18.0	13.5
Corrected cumulative number of infections by 10 May	177,242	88,621	59,081	44,310

**Table 8:** Indicative values of the epidemics in case of the applied control measures. Hospital and ICU bed need at the peak, mortality and the number of recovered people with the expected time it takes to reach 1,000 ICU beds is shown in case our control scenarios. See the corresponding time series in Figure 8.

Transmission Reduction	Reproduction Number	Hospital Bed Need at Peak	ICU Need at Peak	Time to Reach 1000 ICU Beds	Mortality (Pers.)	Recovered (of Tot. Pop.)
25%	1.65	20,973	7225	6 weeks	21,624	58.25%
40%	1.32	7400	2477	10 weeks	12,374	37.37%
50%	1.1	1069	350	-	4447	14.84%
60%	0.9	-	-	-	-	-

**Table 9:** Measures applied in Hungary with the date of introduction.

Date	Measure	Reported Number of Cases at the Time of Introduction
8 March	Banned visits to health care institutions and long-term care facilities	9
9 March	Suspension of Northern Italy flights	12
11 March	Emergency notification	16
12 March	University closures, no entry for non-Hungarian passengers to Hungary from Italy, China, Korea and Iran	19
16 March	School closures	50
17 March	Shortened opening time of shops, ban on events	58
28 March	Stay at home measures	408
4 May	Partial lifting of stay at home measures and opening of restaurants in the countryside (except Pest county where from May 14)	3065
18 May	Lifting of stay at home measures and opening of shops and outdoor areas of restaurants in Budapest	3556
